# Evaluation of Phenolic Root Exudates as Stimulants of Saptrophic Fungi in the Rhizosphere

**DOI:** 10.3389/fmicb.2021.644046

**Published:** 2021-04-14

**Authors:** Anna Clocchiatti, S. Emilia Hannula, Marlies van den Berg, Maria P. J. Hundscheid, Wietse de Boer

**Affiliations:** ^1^Netherlands Institute of Ecology (NIOO-KNAW), Wageningen, Netherlands; ^2^Soil Biology Group, Wageningen University, Wageningen, Netherlands

**Keywords:** phenolic acids, root exudates, saprotrophic fungi, fungal biomass, fungal community, *Trichoderma*, *Fusarium*

## Abstract

The rhizosphere microbial community of crop plants in intensively managed arable soils is strongly dominated by bacteria, especially in the initial stages of plant development. In order to establish more diverse and balanced rhizosphere microbiomes, as seen for wild plants, crop variety selection could be based on their ability to promote growth of saprotrophic fungi in the rhizosphere. We hypothesized that this can be achieved by increasing the exudation of phenolic acids, as generally higher fungal abundance is observed in environments with phenolic-rich inputs, such as exudates of older plants and litter leachates. To test this, a rhizosphere simulation microcosm was designed to establish gradual diffusion of root exudate metabolites from sterile sand into arable soil. With this system, we tested the fungus-stimulating effect of eight phenolic acids alone or in combination with primary root metabolites. Ergosterol-based fungal biomass measurements revealed that most phenolic acids did not increase fungal abundance in the arable soil layer. These results were supported by comparison of fungal biomass in the rhizosphere of wild type *Arabidopsis thaliana* plants and mutants with altered phenolic acid metabolism. Salicylic acid was the only phenolic acid that stimulated a higher fungal biomass in the arable soil layer of microcosms, but only when combined with a background of primary root metabolites. However, such effect on rhizosphere fungi was not confirmed for a salicylic acid-impaired *A. thaliana* mutant. For three phenolic acid treatments (chlorogenic acid, salicylic acid, vanillic acid) fungal and bacterial community compositions were analyzed using amplicon sequencing. Despite having little effect on fungal biomass, phenolic acids combined with primary metabolites promoted a higher relative abundance of soil-borne fungi with the ability to invade plant roots (*Fusarium, Trichoderma* and *Fusicolla* spp.) in the simulated rhizosphere. Bacterial community composition was also affected by these phenolic acids. Although this study indicates that phenolic acids do not increase fungal biomass in the rhizosphere, we highlight a potential role of phenolic acids as attractants for root-colonizing fungi.

## Introduction

The rhizosphere, the soil volume surrounding plant roots, is a hotspot for microbial activity (Pausch and Kuzyakov, 2018), harboring saprotrophic fungi, alongside bacteria and mycorrhizal fungi (Buée et al., 2009; van der Putten et al., 2016; Hugoni et al., 2018). Rhizosphere saprotrophic fungi can provide multiple services to the plant, such as promotion of plant growth and immunity (Koike et al., 2001; Kohler et al., 2007; Yadav et al., 2011; Naznin et al., 2014; Xia et al., 2019), as well as suppression of infection by soil-borne fungal pathogens. The presence of active saprotrophic fungi can limit root infection by soil-borne pathogens by increasing the competition for resources in the rhizosphere or by direct inhibitory activities such as antibiosis or mycoparasitism (Punja and Utkhede, 2003; Xiong et al., 2017; Latz et al., 2018). Moreover, rhizosphere saprotrophic fungi can influence beneficial rhizosphere bacteria and mycorrhizal fungi, hence indirectly influencing plant performance (Kohler et al., 2007; Saldajeno et al., 2008; de Boer et al., 2015; Qin et al., 2017; Deveau et al., 2018).

Intensively managed arable soils usually harbor low saprotrophic fungal biomass (Djajakirana et al., 1996; de Vries and Bardgett, 2012), which is also reflected in low activity of saprotrophic fungi in the rhizosphere of crop plant seedlings (Hünninghaus et al., 2019). Low fungal biomass in arable soils can be attributed to a combination of factors such as low input of organic resources (van der Wal et al., 2006; Clocchiatti et al., 2020), use of chemical fungicides (Duah-Yentumi and Johnson, 1986; Shao and Zhang, 2017) and intensive tillage. Hence, strategies that promote high saprotrophic fungal biomass and activity in the rhizosphere of crops could be important to enhance the sustainability of agricultural cultivation.

Saprotrophic fungal abundance in the rhizosphere largely depends on the availability of appropriate energy sources. The amendment of arable soils with organic substrates, such as manure, straw, cover crop remainders or sawdust, can be used to increase fungal biomass in the bulk soil (Lucas et al., 2014; Arcand et al., 2016; Clocchiatti et al., 2020) and also may increase fungal abundance and activity in the rhizosphere (Hannula et al., 2012). During the growth of crop plants, rhizodeposits are the main organic input into soil in conventional monocultures. However, high fungal biomass is observed in the crop rhizosphere only at late phenological stages, such as flowering and senescence, and in perennial crops (Hannula et al., 2010; Tavi et al., 2013; Pausch et al., 2016). This can be attributed to the deposition of a larger proportion of cellulose-rich root debris released by older roots (Dennis et al., 2010; Pausch and Kuzyakov, 2018). Moreover, older plants tend to exude a higher proportion of soluble secondary metabolites, including phenolic acids, as compared to younger plants (Gransee and Wittenmayer, 2000; Chaparro et al., 2013; Iannucci et al., 2013; Zhalnina et al., 2018). Hence, in order to increase the contribution of saprotrophic fungi in the rhizosphere of crop seedlings, an approach involving manipulation of the quantity and quality of rhizodeposits may prove useful. For example, the release of fungus-stimulating root exudates could be used as selection criterion for breeding crop varieties.

Phenolic acids exert a strong selection on rhizosphere microbial communities. As phenolic acids are toxic to microbial cells at relatively low concentrations, they act as a deterrent for sensitive microbial groups, whilst they favor those groups that possess metabolic pathways for phenolic degradation (Fierer et al., 2005; Pumphrey and Madsen, 2008). The ability to degrade phenolic acids is very common among saprotrophic fungi, as it is essential to consume or tolerate free aromatic compounds released during lignocellulose depolymerization (Cain et al., 1968; Sampedro et al., 2004; Mäkelä et al., 2015). When soils receive inputs rich in simple phenolics, saprotrophic fungi are among the main microbial utilizers of such compounds (Waldrop and Firestone, 2004; Brant et al., 2006) and this can lead to a large increase in fungal abundance (Zhou et al., 2012; Suseela et al., 2016; Wang et al., 2016). This suggests that phenolic acids in root exudates could promote the colonization by saprotrophic fungi also in the rhizosphere of crop plants. However, as certain groups of bacteria can also utilize phenolic acids (Blum and Shafer, 1988), the relative stimulation of fungi by phenolic root exudates may depend on soil edaphic factors.

The objective of this study was to investigate the effect of phenolic acids on growth and composition of saprotrophic fungi in simulated and real rhizospheres in an arable soil. The first part of this study was conducted with a microcosm system that simulates root exudation via diffusion of artificial metabolite solutions. This rhizosphere simulation system was used to investigate 1) if diffusion of distinct phenolic acid compounds, alone or in combination with primary root exudate metabolites, increases fungal biomass and 2) if this modulates the community composition of fungi and bacteria. In the second part of this study, fungal development in the rhizosphere of *Arabidopsis thaliana* was compared between wild types and mutant lines *pdr2* and *sid2*, altered in the proportion of exuded phenolic acids and biosynthesis of salicylic acid, respectively. The effect of altered exudation of phenolic compounds on the rhizosphere fungal biomass was tested for two plant developmental stages. We hypothesized that the presence of phenolic acids in root exudates promotes the growth of saprotrophic fungi and increases their competitive ability to utilize other energy sources (i.e., primary metabolites) in the rhizosphere.

## Materials and Methods

**FIGURE 1:**
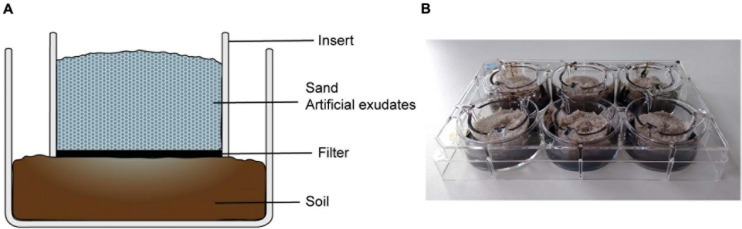
Setup of rhizosphere simulation microcosms in a two-compartment well with insert. **(A)** In a microcosm, the lower compartment contained arable soil, the upper compartment (insert) was filled with sterile sand mixed with artificial exudates. A permeable filter separates the compartments, which allows the diffusion of artificial exudates from the sand in the soil, simulating root exudation. **(B)** Setup of six microcosms in a six-well plate with inserts.

The study comprised of two experiments. The first experiment (Exp. 1) was carried out in two-compartment microcosms, which were used to simulate diffusion of root exudates into arable soil (Figure 1). Using this set-up, we investigated the effect of eight phenolic acids on abundance and community composition of fungi and bacteria. In the second experiment (Exp. 2), *A. thaliana* mutant lines with altered phenolics exudation patterns and wild-type *A. thaliana* lines were grown in an arable soil. Fungal biomass was measured in the rhizosphere of these plants at two developmental stages.

### Characteristics of Soil and Sand

Two batches of soil were sampled in October 2016 and July 2019 and were used for Exp. 1 and 2, respectively. The soil was sampled at the experimental farm of Wageningen University & Research located in Vredepeel (N 51 32 19, E 5 51 05, the Netherlands) from the top 10 cm soil layer, within patches of bare soil in between triticale (first batch) and maize plants (second batch), in a plot that was subjected to conventional agricultural management practices. The soil was sandy and had a relatively high organic matter content (6.3%). For more details on soil management and soil characteristics see Quist et al. (2016) and Clocchiatti et al. (2020). The soil samples were sieved through a 4-mm mesh and stored at 4°C until use. Moreover, acid-washed quartz sand (granulation 0.1–0.5 mm; Honeywell Speciality Chemicals Seelze GmbH, Seelze, Germany) was used in Exp. 1. Before use, the sand was autoclaved (121°C for 20 min) and dried under sterile conditions.

### Preparation of Artificial Exudates Solutions

The study included eight water-soluble phenolic acid compounds that are commonly found in root exudates (Table 1): vanillic acid, syringic acid, gallic acid, salicylic acid, chlorogenic acid, nicotinic acid, ferulic acid and cinnamic acid. A suspension containing 0.5 mg C ml^–1^ in 50 ml sterile demi-water was prepared for each compound. The pH of all suspensions was adjusted to 6 with NaOH/HCl and the suspensions were subjected to sonication for 20 min at 47 kHz, in order to facilitate the dissolution of the phenolic compounds. Half of each phenolic acid solution was filter-sterilized and stored at 4°C and used in Exp. 1. The remaining solution was mixed with a stock solution of primary root exudate metabolites (PM) prepared according to Griffiths et al. (1998) (Table 1). The concentration of PM in the working solution was 13.4 M glucose, 13.4 mM fructose, 13.4 mM sucrose, 6.7 mM succinic acid, 6.7 mM malic acid, 3.35 mM arginine, 3.35 mM serine and 3.35 mM cysteine. Solutions containing PM and a phenolic acid had a total carbon content of 5.5 mg C ml^–1^. These PM + phenolic acid solutions were filter-sterilized and stored at 4°C until use in Exp 1.

**TABLE 1:** Phenolic acids and primary metabolites used in Exp. 1.

Compound	Concentration in AE solution (mg ml^–1^)	Concentration in sand (mg C g^–1^)	References	Source
**Phenolic acids**	-	0.1		
Vanillic acid	0.875	0.1	Shukla et al., 2011; Iannucci et al., 2013; Ray et al., 2018; Zhalnina et al., 2018	Sigma-Aldrich, H36001
Syringic acid	0.917	0.1	Shukla et al., 2011; Iannucci et al., 2013; Zhalnina et al., 2018	Sigma-Aldrich, 86230
Gallic acid	0.642	0.1	Shukla et al., 2011; Ray et al., 2018	Sigma-Aldrich 48630
Salicylic acid	0.821	0.1	Badri et al., 2013; Shalaby and Horwitz, 2015; Gao et al., 2018; Zhalnina et al., 2018	Sigma-Aldrich S-7401
Chlorogenic acid	0.922	0.1	Shukla et al., 2011; Shalaby and Horwitz, 2015	Merck 1.59619.0001
Nicotinic acid	0.854	0.1	Shukla et al., 2011; Zhalnina et al., 2018	Sigma-Aldrich 72309
Ferulic acid	0.808	0.1	Badri et al., 2013; Iannucci et al., 2013; Shalaby and Horwitz, 2015; Gao et al., 2018; Ray et al., 2018	Sigma-Aldrich 128708
Cinnamic acid	0.685	0.1	Shukla et al., 2011; Shalaby and Horwitz, 2015; Gao et al., 2018	Sigma-Aldrich C80857
**Primary metabolites**		1	Griffiths et al., 1998; Shukla et al., 2011	
Glucose	2.42	0.19		Roth 6639
Fructose	2.66	0.19		Sigma-Aldrich F-0127
Sucrose	4.60	0.39		Sigma-Aldrich S-9378
Succinic acid	0.79	0.06		Sigma-Aldrich S3674
Malic acid	0.90	0.06		Sigma-Aldrich M-9138
Arginine	0.58	0.05		Sigma-Aldrich A-8094
Serine	0.35	0.02		Sigma-Aldrich S-5511
Cysteine	0.41	0.02		Sigma-Aldrich C7352

*For each compound are displayed: the concentration used for preparing the artificial exudates (AE) solution, the amount of added carbon per gram of sand, references to literature reporting the presence of each compound in root exudates and the product used in this study.*

### Setup of Two-Compartment Rhizosphere Simulation Microcosms

Before the start of Exp. 1, the arable soil was adjusted to 75% water holding capacity (WHC) by adding sterile demi-water and acclimatized at 21°C in a dark climate chamber for 2 days. Six-well plates (6-well CELLSTAR plates and ThinCert cell culture inserts, Greiner Bio-One B.V., the Netherlands) were used for setting up microcosms. Each microcosm was set up in a well containing an insert, so that it had a lower compartment and an upper compartment, separated by a permeable polyethylene terephthalate membrane (Figure 1). The membrane had 0.4 μm pores, which enabled the diffusion of dissolved molecules between the two compartments, but hampered the migration of soil microbes. In all microcosms, the lower compartment was filled with soil equivalent to 5 g dry weight. The insert was then placed on top of the soil layer, taking care that the filter membrane adhered to the soil. The upper compartment was filled with 5 g of pure sterilized sand mixed with a solution of either phenolic acids only, PM or PM + phenolic acid. The sand had a moisture of 75% WHC, which was obtained by adding the described metabolites solutions (0.2 ml g^–1^ sand) and demi-water (7.4 μl g^–1^ sand). All the materials in the upper compartment were sterile, thus the soil was the only source of microbes in the microcosm. The metabolites added in the sand compartment diffuse into the soil through the filter, simulating root exudation.

The sand in each unit received either a single phenolic acid, equivalent to 0.1 mg C g^–1^ sand, or a phenolic acid combined with PM, containing in total 1.1 mg C g^–1^ sand. In the latter mixture, the proportion of a phenolic acid was ca. 10% of the total C, which resembles the proportions observed in root exudates (Narasimhan et al., 2003). The experiment had two separate controls, namely sand receiving sterile water only and sand receiving PM only. In total, 18 types of solutions were used (eight phenolic acids × with/without PM + 2 controls). Each treatment was applied in four replicate microcosms, making up a total of 72 microcosms across 12 six-well plates. The solutions were distributed over the plates according to a complete randomized block design.

### Incubation and Sampling of the Two-Compartment Rhizosphere Simulation Microcosms

Each plate was sealed with surgical tape (Micropore, 3M, Minnesota, United States), which allowed gas exchange, while limiting water evaporation. The two-compartment microcosms were incubated for 2 weeks in a dark climate chamber at 21°C. After 2 weeks, the sand and soil were sampled separately from all microcosms. For sand, 0.5 g samples were obtained from a 2 mm layer close to the membrane filter. These samples were used for ergosterol-based fungal biomass determination, while the rest of the sand present in the inserts was used to measure the moisture content. Similarly, 1 g samples of soil were taken from the 2 mm layer right below the filter. Half of this sample was used to determine fungal biomass with ergosterol and half was stored at −20°C to be used for DNA extraction. Samples for ergosterol extraction were stored in methanol KOH 4% at −20°C. Soil moisture was determined by using the rest of the soil in the lower compartment. Moisture content of sand and soil were measured as weight loss following oven-drying at 105° C overnight.

### Fungal Biomass

Ergosterol was extracted from soil and sand samples as described by de Ridder-Duine et al. (2006). Briefly, the extraction method is based on sonication, followed by heat treatment and a subsequent alkaline hydrolysis of esterified ergosterol, aided by mechanical shaking. The ergosterol concentration was quantified in the final methanol-based extract by LC-MS-MS (UHPLC 1290 Infinity II, Agilent Technologies and 6460 Triple Quad LC-MS, Santa Clara, California, United States).

### Fungal and Bacterial Community Structure and Bacterial Abundance

DNA was extracted from 0.25 g soil using DNeasy PowerSoil Pro Kit (Qiagen, Germany) according to the manufacturer’s instructions. DNA was extracted for a selection of soil samples in Exp. 1, namely from microcosms of vanillic acid -, salicylic acid -, chlorogenic acid - and water treatments, both with and without PM. This selection covered phenolic acids with and without fungal stimulation effects in Exp. 1. Vanillic acid was included in the selection to allow comparisons with the numerous studies that include this compound as a representative of phenolic acids (Waldrop and Firestone, 2004; di Lonardo et al., 2017; Chen et al., 2018). The DNA was subjected to bacterial and fungal amplicon sequencing using Illumina MiSeq PE250, which was performed by McGill University and Génome Québec Innovation Centre, Montréal, Canada, with primers Eub515f/806r (Caporaso et al., 2011) and ITS4r/9f (Ihrmark et al., 2012), for bacteria and fungi, respectively.

The 16S rDNA gene copy number was determined by qPCR for the control soil samples and those treated with vanillic - and salicylic acid, both with and without PM. The qPCR analysis was performed on a Rotor-Gene Q Real-time PCR cycler (Qiagen). The mix comprised 0.5 μl of each primer Eub 338/518 10 μM, 7.5 μl iTaq Universal SYBR green supermix (Bio-Rad Laboratories, California, United States), 2.5 μl Nuclease-Free water (Sigma-Aldrich, Missouri, United States) and 4 μl DNA 2.5 ng μl^–1^. The qPCR cycling program was 3 min at 95°C followed by 40 cycles of 30 s at 95°C, 30 s at 53°C and 30 s at 72°C. Two standard curves were established for each run (0.23 ng μl^–1^, 0.023 ng μl^–1^, 0.0023 ng μl^–1^, 0.00023 ng μl^–1^, 0.000023 ng μl^–1^, 0.0000023 ng μl^–1^) with a M13 plasmid containing the 16S region obtained from a *Collimonas* pure culture.

### *Arabidopsis thaliana* Varieties

Four *A. thaliana* lines were used in Exp. 2, of which two were wild-type accessions (Col-0 and Col-8) and represented the controls, whereas two mutant lines (*sid2* and *pdr2*) were used to test the response of fungal biomass to altered exudate composition. *Sid2* (salicylic acid induction deficient 2) is a mutant impaired in salicylic acid production. As a consequence of a defect in the isochorismate synthase 1 (ICS1) gene, it fails to accumulate salicylic acid in its tissues (Wildermuth et al., 2001). *Pdr2* (pleiotropic drud resistance 2) is a knockdown mutant for the ATP-binding cassette transporter G30 (ABCG30), which is involved in root exudation. Badri et al. (2009) showed that this results in an altered exudation pattern with higher proportion of phenolic acids, among other secondary metabolites, and a lower proportion of sugars. Seeds of the line Col-0 were obtained from seeds available in house, whereas seeds of Col-8, *sid2* and *pdr2* were obtained from Nottingham *Arabidopsis* Stock Centre (stock IDs N60000, N16438, N66055). Seeds were propagated by growing each line in potting soil, under protective plastic covers, to prevent cross-pollination. Seeds were collected and stored at 15°C until use. Before the start of Exp. 2, seeds of each line were surface-sterilized by soaking in ethanol 70% for 3 min, followed by rinsing three times with sterile-demi water. After that, seeds were laid on wet filters in petri dishes and vernalized by incubation at 4°C for four nights.

### Setup of the Bioassay

Fifty pots (polyethylene, Ø 14.5 cm × 11.5 cm) were prepared for Exp. 2 as follows. Vredepeel soil was brought to 60% WHC and mixed with 0.6 g kg^–1^ of a commercial NPK fertilizer (Tuinmest 12-10-18, Pokon Naturado, the Netherlands), corresponding to the addition of 72 mg N kg^–1^, 60 mg P kg^–1^, 84 mg K kg^–1^. Each pot was filled with 1.4 kg soil and sown with 80 *A. thaliana* seeds of the same line. These were distributed over 20 spots (interspace 1 cm) on the soil surface. Each spot contained four seeds. The pots were incubated in a random arrangement (CRBD) in a climate chamber at 21°C, with photoperiod 12:12. 10 days after the start of the growth period, all but one seedling per spot were removed. Hence, a maximum of 20 seedlings were grown in each pot. The experiment comprised of ten replicate pots for each *A. thaliana* line and ten un-planted control pots. Of these, five replicates for each line were harvested 33 days after the start of the experiment, few days before the bolting stage. The other five replicates were harvested after 50 days of incubation, during the flowering stage.

### Harvesting of Plants and Sampling

At both developmental stages, plant and soil samples were obtained as follows. Excess soil was removed from the roots by shaking and the soil adhering to the roots was collected by brushing. The rhizosphere soil of all plants in a pot was pooled to form one composite sample. Of this, 1 g was stored in methanol KOH 4% at −20°C until ergosterol extraction. In addition to this, 1 g of bulk soil was sampled from each pot and stored in the same way for ergosterol extraction. Ergosterol was extracted and quantified as described for Exp. 1. Roots and shoots were kept separate from each other but pooled per pot, frozen, freeze-dried and used as a measure of total aboveground and belowground dry biomass produced in each pot.

### Statistical and Bioinformatic Analysis

The statistical analyses were carried out in R version 3.4.0. For Exp. 1, three-way ANOVA was performed to compare the ergosterol levels across microcosms that received different types of metabolites. The model comprised the type of phenolic acid, presence/absence of PM and compartment (soil or sand) as factors. The model included block as a factor as well. The assumptions of equality of variances and normality were verified for the model. The ergosterol concentration was compared between each metabolite type and the control by applying planned contrasts to the three-way ANOVA model (R package lsmeans), combined with Dunn–Šidák correction of *p*-values for multiple comparisons (Šidák, 1967). The same method was used to perform a two-way ANOVA, in order to compare 16S copy number as measured in the soil in microcosms receiving phenolic acids with and without PM.

Two-way ANOVA models were carried out for Exp. 2, after checking the assumptions of homoscedasticity and normality. ANOVA models were used for comparing four *A. thaliana* lines at two developmental stages, with block as a random factor, for the following variables: ergosterol concentration in the rhizosphere, ratio of ergosterol in the rhizosphere and bulk soil, aboveground and belowground plant biomass in a pot.

The raw sequencing data contained 2 767 341 sequences for fungi and 374 216 sequences for bacteria. The ITS2 region was extracted with ITSxpress from fungal sequences (Rivers et al., 2018) before analyzing the data with R package DADA2, whereas 16S data was processed directly using the DADA2 pipeline without an extra filtering step. Sequences were quality filtered (maxEE = 2, truncQ = 2, only for bacteria: truncLen = 240), paired-end reads were merged, chimeric sequences were removed, sequencing errors modelled and finally sequence variants (SVs) were identified by the DADA2 algorithm (Callahan et al., 2016). Taxonomy was assigned using the RDP classifier based on the UNITE v2019 database (Abarenkov et al., 2010) for fungi and SILVA v132 for bacteria. After removing non-fungal and non-bacterial sequences from each dataset, the fungal dataset resulted in 1 046 SVs, whereas the bacterial dataset counted 5 992 SVs. Fungal guilds were assigned to each fungal SV when possible, using the FUNGuild database v1.1 (Nguyen et al., 2016). In addition to guild, information about the growth mode and life history traits were retained in the analysis, in order to distinguish types of saprotrophic fungi (e.g., yeasts, filamentous microfungi and soft rot fungi). Permutational multivariate analysis of variance (PERMANOVA) was used to determine the effect of phenolic acids with and without a background of PM, after checking the homogeneity of multivariate variance (vegan, PERMDISP, Anderson and Walsh, 2013). Differences in relative abundance of fungal taxa between soils treated with different metabolites were analyzed at phylum, class and genus level and for fungal functional guilds. For both fungal and bacterial communities Chao1 and Shannon indexes were calculated. Differences in diversity, as well as in relative abundance were analyzed with two-way ANOVA models, after verification of normality and homoscedasticity of each model. Planned contrasts between each metabolite and the control were performed for ANOVA models to extract the simple effect of a phenolic acid with or without PM. *P*-values were corrected with the Dunn–Šidák method. Differences in bacterial community composition between soil treated with salicylic acid + PM as compared to PM alone were highlighted by differential abundance analysis (R package DESeq2, wald test, *p* < 0.01).

## Results

### Fungal Biomass

**FIGURE 2:**
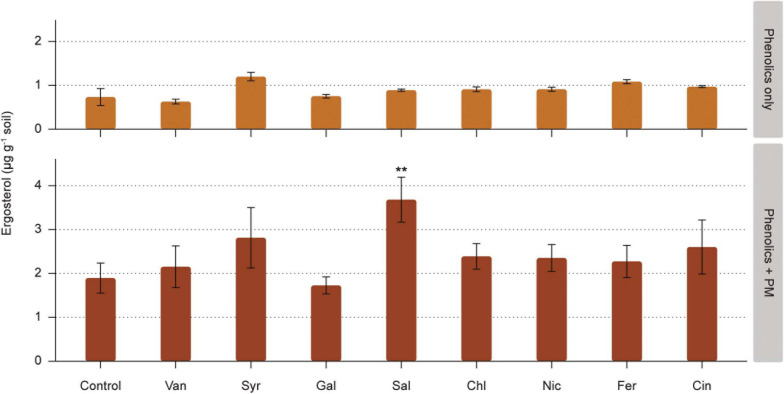
Effect of phenolic acids on fungal biomass, as measured in the soil compartment of rhizosphere simulation microcosms. Phenolic acids were vanillic acid (Van), syringic acid (Syr), gallic acid (Gal), salicylic acid (Sal), chlorogenic acid (Chl), nicotinic acid (Nic), ferulic acid (Fer), and cinnamic acid (Cin). Phenolic acids were added alone or with a background of soluble primary metabolites (PM). Significant differences to the control (water and PM only, respectively) are shown for phenolic acids without and with PM (** 0.01 > *p* > 0.001).

The ergosterol concentration in the arable soil layer of the microcosms (Exp. 1) increased in response to the presence of primary root exudate metabolites (PM) in the upper sand layer (ANOVA, *df* = 1, *F* = 186.6, *p* < 0.001, Supplementary Table 1A). Presence of phenolic acids in the upper sand layer had a smaller yet significant effect on ergosterol concentration (ANOVA, *df* = 8, *F* = 3.2, *p* < 0.01). However, the effect of phenolic acids was dependent on the presence of PM (seen as interaction PM x phenolic acid, Supplementary Table 1A). Especially, adding salicylic acid together with PM resulted in an extra increase of ergosterol compared to the PM-only control (*p* < 0.01) while salicylic acid alone did not increase ergosterol concentration in the soil as compared to the water only control (Figure 2). When compared directly with the controls, none of the other phenolic acids with or without PM had a significant effect on the ergosterol concentration in the soil layer.

Despite the small pore size (0.4 μm) of the insert membrane, fungi in the soil compartment were able to reach the sterile upper sand compartment especially in the treatments in which PM were added together with phenolic acids (Supplementary Figure 1). In the sand compartment, salicylic, nicotinic and ferulic acid added together with PM increased ergosterol concentration more than PM added alone (Supplementary Figure 1).

**FIGURE 3:**
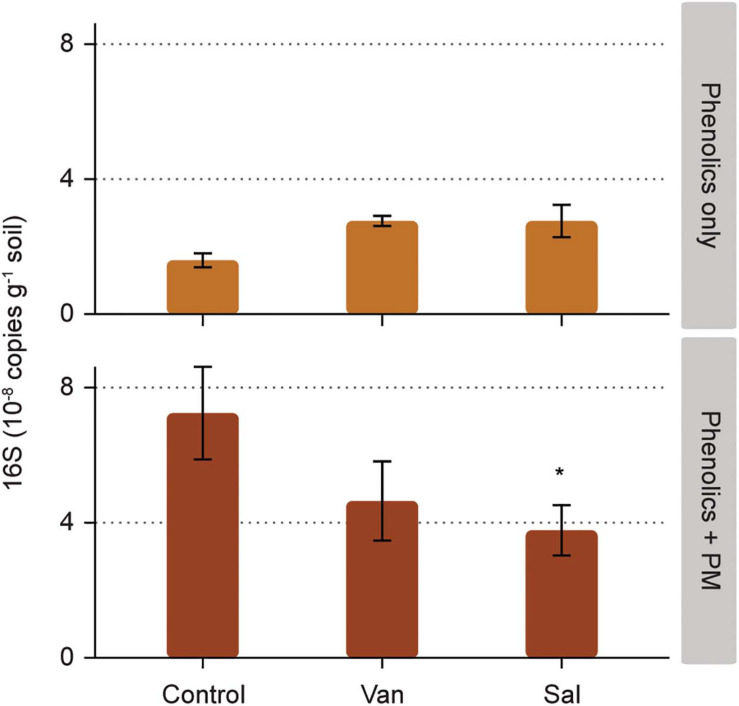
Effect of phenolic acids on bacterial numbers, as measured in the soil compartment of rhizosphere simulation microcosms. Phenolic acids were vanillic acid (Van) and salicylic acid (Sal), applied alone and with a background of soluble primary metabolites (PM). Significant differences to the control (water and PM only, respectively) are shown for phenolic acids without and with PM (* 0.05 > *p* > 0.01).

Bacterial numbers in the soil were not significantly affected by the addition of vanillic acid and salicylic acid alone (Figure 3 and Supplementary Table 1). The addition of PM alone resulted in a significant increase of bacterial numbers (measured as 16S rDNA copies) in the soil, as compared to the control with addition of only water (*p* < 0.05; Figure 3). Salicylic acid combined with PM had a lower bacterial abundance as compared to the addition of PM only (*p* < 0.05, Figure 3 and Supplementary Table 1).

**FIGURE 4:**
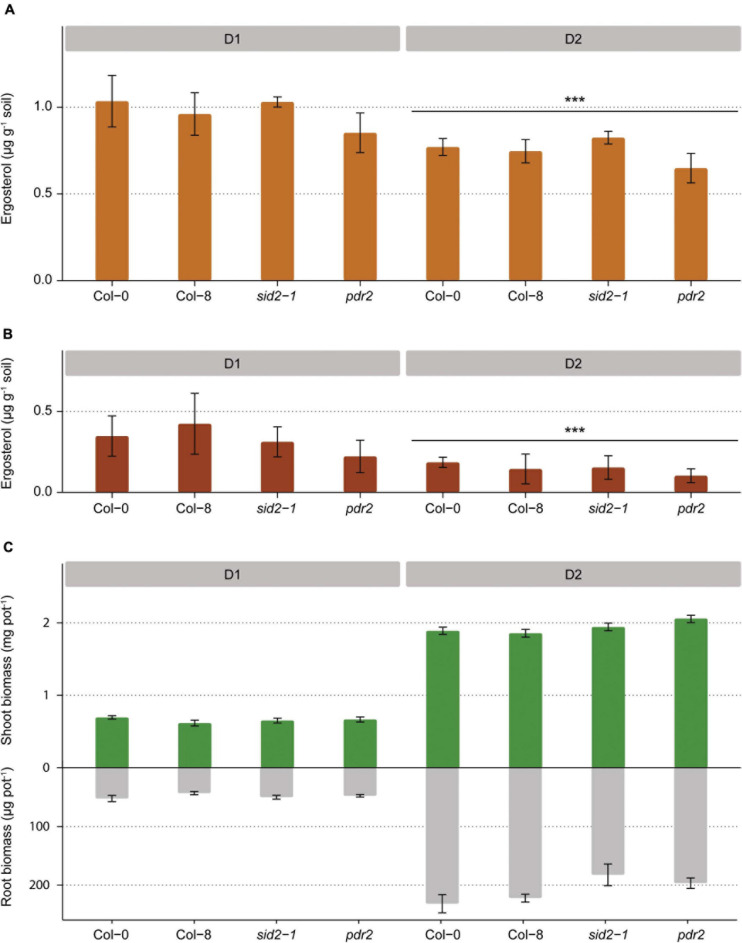
Fungal biomass in the rhizosphere soil of four *Arabidopsis* lines at two developmental stages (D1 and D2) measured as ergosterol concentration **(A)** and increase in ergosterol concentration from the level measured in the bulk soil **(B)**. Aboveground and belowground dry plant biomass collected from each pot for four *Arabidopsis* lines at two developmental stages **(C)** (*** *p* < 0.001).

In Exp. 2, ergosterol concentrations were higher in the rhizosphere than the bulk soil for all *A. thaliana* accessions (Tukeys’ test, *p* < 0.001 for both developmental stages; Figure 4B). Furthermore, ergosterol content was higher in the rhizosphere of plants at the first developmental stage (D1) than at the second stage (D2) (Tukeys’ test, *p* < 0.001; Figures 4A,B). No differences were found in rhizosphere ergosterol levels among the different *A. thaliana* lines (Figure 4 and Supplementary Table 2) and the four *A. thaliana* lines also produced comparable belowground and aboveground biomass (Figure 4C).

### Fungal and Bacterial Community Structure

**FIGURE 5:**
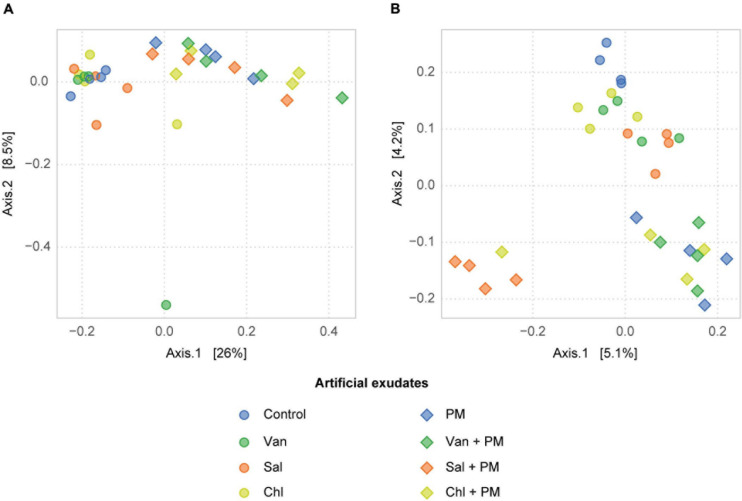
Effect of phenolic acids on fungal **(A)** and bacterial **(B)** communities in the arable soil layer of rhizosphere simulation microcosms. PCoA ordination plot based on Bray-Curtis distances. Four replicates are shown for the control, vanillic acid (Van), salicylic acid (Sal) and chlorogenic acid (Chl), applied alone (phenolics only) and with primary metabolites (phenolics + PM).

**FIGURE 6:**
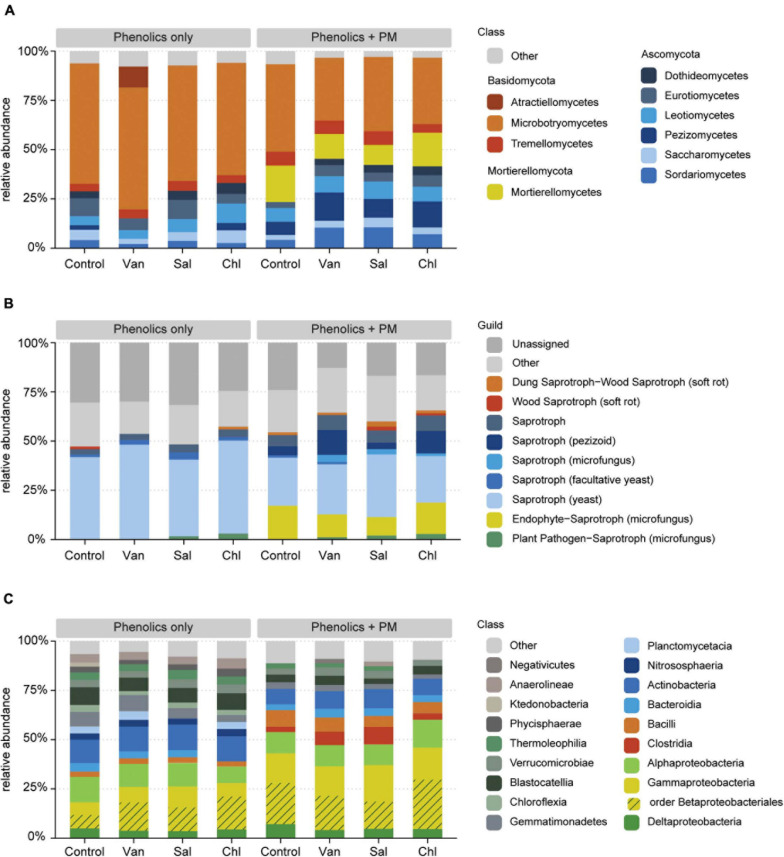
Effect of phenolic acids (phenolics only) and phenolic acids combined with primary metabolites (phenolics + PM) on the relative abundance of fungal classes **(A)**, fungal functional groups **(B)**, and bacterial classes **(C)**, as detected in the arable soil compartment of rhizosphere simulation microcosms. Classes constituting < 2% of each community are displayed as “Other.” The result is shown for the control, vanillic acid (Van), salicylic acid (Sal) and chlorogenic acid (Chl).

**FIGURE 7:**
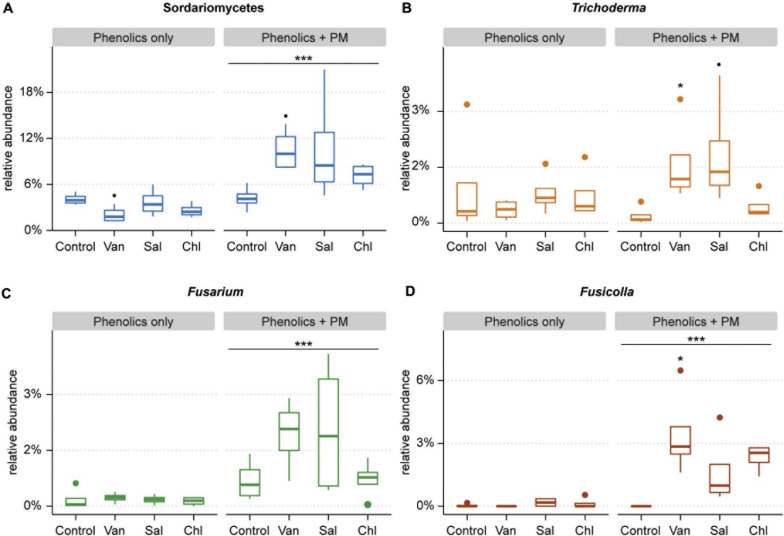
Relative abundance of Sordariomycetes **(A)** and the three most abundant sordariomycetal genera: *Trichoderma* **(B)**, *Fusarium* **(C)**, and *Fusicolla* **(D)**, as found in the soil compartment of in rhizosphere simulation microcosms treated with vanillic acid (Van), salicylic acid (Sal), chlorogenic acid (Chl) or control, with and without primary metabolites (PM). The main effect of primary metabolites is shown, as well as the effect of a phenolic acid as compared to the control (^∙^0.1 > *p* > 0.05, *0.05 > *p* > 0.01, ***p < 0.001).

In the rhizosphere simulation microcosms, the soil fungal community was dominated by Basidiomycota, in particular Microbotryomycetes. The analysis of fungal communities in control soil and in soil receiving vanillic, salicylic and chlorogenic acid, with and without PM, revealed that the application of PM, but not of the tested phenolics, significantly affected the composition of the fungal community (PERMANOVA, *df* = 1, *F* = 0.38, *R*^2^ = 0.19, *p* < 0.001; Figure 5A and Supplementary Table 3). In treatments with PM, increases in relative abundances were detected for Mortierellomycota (*df* = 1, *F* = 64.1, *p* < 0.001), Ascomycota (*df* = 1, *F* = 5.7, *p* < 0.05) and, among Basidiomycota, for Tremellomycetes (*df* = 1, *F* = 9.0, *p* < 0.01) (Figures 6A, 7A). Among functional guilds, presence of PM resulted in an increase of filamentous fungi classified as saprotrophs (microfungi, pezizoid, soft rot; *df* = 1, *F* = 12.4, *p* < 0.01, Figure 6B) and of fungi classified as endophyte-saprotroph (*df* = 1, *F* = 63.7, *p* < 0.001, Figure 6B).

Despite the lack of overall effects of the three analyzed phenolic acids treatments on fungal community structure, specific effects were apparent. Treatments with vanillic acid + PM and salicylic acid + PM compared to PM alone had a higher proportion of Sordariomycetes (*p* = 0.061 and *p* = 0.053, respectively) (Figures 6A and 7A). The most abundant Sordariomycetes were *Trichoderma* spp., *Fusarium* spp. and *Fusicolla* spp. (Figures 7B–D). When combined with PM, vanillic acid and salicylic acid promoted *Trichoderma* spp. as compared to PM alone (*p* < 0.05 and *p* = 0.07, respectively). Vanillic acid + PM and salicylic acid + PM had a higher average relative abundance of *Fusarium* spp. as compared to PM only, however, this effect was not significant (*p* = 0.1 and *p* = 0.3, respectively). *Fusicolla* spp. increased as compared to PM alone, with vanillic acid + PM and chlorogenic acid + PM (*p* < 0.001 and *p* < 0.05, respectively).

Bacterial community composition was affected by addition of PM and by the three analyzed phenolic acid treatments, (Figure 5B and Supplementary Table 3B). In particular, PM increased γ-Proteobacteria (*df* = 1, *F* = 32.0, *p* < 0.001), Bacilli (*df* = 1, *F* = 40.0, *p* < 0.001) and Clostridia (*df* = 1, *F* = 14.8, *p* < 0.001) (Figure 6C). The phenolic acids examined altered the bacterial community composition both alone (*df* = 3, *F* = 0.04, *R*^2^ = 0.21, *p* < 0.01) and with a background of PM (*df* = 3, *F* = 1.17, *R*^2^ = 0.23, *p* < 0.01). In particular, salicylic acid + PM had the largest dissimilarity from the control with PM only (Figure 5B). Therefore, this treatment was analyzed in more detail. Differential abundance analysis (wald test, *p* < 0.01) showed that 210 SVs were overrepresented in the bacterial community affected by salicylic acid + PM, as compared with PM only. Conversely, 123 SVs were overrepresented in the community affected by PM only, as compared to salicylic acid + PM (Supplementary Figure 3). Bacteria favored by salicylic acid + PM or by PM alone were distributed across all the bacterial classes, included α-, γ-, δ-Proteobacteria, Bacilli, Clostridia, Bacteroidia and Actinobacteria (Supplementary Figure 3).

## Discussion

### Effect of Phenolic Acids in Root Exudates on Abundance and Community Composition of Fungi and Bacteria

The effect of individual phenolic acids on fungal biomass was tested in rhizosphere simulation microcosms. We expected that diffusion of phenolic acids from the sterile upper sand layer into arable soil would increase saprotrophic fungal abundance. Yet, we detected no significant effect of added phenolic acids on fungal biomass in the arable soil. Diffusion of primary root exudate metabolites had a strong positive effect of fungal biomass in the arable soil layer, but combination of primary metabolites with phenolic acids did not result in an additional increase, with exception of one phenolic acid, namely salicylic acid. The analysis of fungal sequences revealed that the primary metabolites in artificial root exudates promoted the growth of Mortierellomycota, Ascomycota and basidiomycetal yeasts in the simulated rhizosphere microcosms. This is in line with what is often observed in rhizospheres and reflects the ability of many fungi of these groups to utilize easy degradable carbon compounds (Porras-Alfaro et al., 2011; Kazerooni et al., 2017; Hugoni et al., 2018).

The absence of selective stimulation by most phenolic acids suggests that these compounds were not preferentially consumed by fungi and did also not increase their competitive ability in the simulated rhizosphere. Indeed, previous studies show that both soil fungi and bacteria can metabolize phenolic acids (Blum and Shafer, 1988; Waldrop and Firestone, 2004; di Lonardo et al., 2017; Zwetsloot et al., 2020), although in some cases phenolic acids stimulated fungi to a larger extent than bacteria (Zhou et al., 2012; Zhou and Wu, 2013; Suseela et al., 2016). These differential responses can be ascribed to the observation that the effect of phenolic acids on fungi and bacteria appear to be concentration dependent. In particular, fungi become dominant in soils receiving high loads of simple phenolics (Blum and Shafer, 1988; Zhou and Wu, 2013). In the study of di Lonardo et al. (2017), a stimulation of fungal abundance was observed in an ex-arable soil at a high concentration of vanillic acid (0.8 mg C g^–1^) and only in combination with supplemental nitrogen, but not at a low vanillic acid concentration (0.1 mg C g^–1^). In monocropped soils, phenolic acids are usually found at concentrations ranging from 0.02 to 0.2 mg C g^–1^ soil (Zhou et al., 2012). In our study, phenolic acids were added at a concentration of 0.1 mg C g^–1^ soil and represented ca. 10% of the total C when mixed with primary metabolites, as based on the proportion of phenolic acids in root exudates reported by Narasimhan et al., 2003. Hence, the low, yet realistic input of phenolic acids into the arable soil may explain the lack of observable effect on fungal biomass.

Interestingly, fungi were able to invade the upper sterile sand compartment when PM were present. Probably 0.4 μm pores were enlarged as a consequence of a partial decomposition of the polyethylene membrane by soil microbes during the 2 weeks of incubation (Gajendiran et al., 2016). This invasion of fungi in the upper compartment could be considered as a simulation of fungi entering the root interior. It was more pronounced in combination with phenolic acids, especially with salicylic, nicotinic and ferulic acid, indicating that fungi were moving toward the source of these phenolic acids.

Fungal biomass was not stimulated in the rhizosphere of *pdr2 Arabidopsis* plants producing higher proportion of phenolic root exudates than wild type plants (Badri et al., 2009). This supports the results of the simulated rhizosphere microcosms. However, it must be noted that the mutation *pdr2* does not target specifically phenolic acids, but also impacts a number of other secondary and primary metabolites (Badri et al., 2009). In order to have better insight in the role of phenolic acids on saprotrophic fungi in the rhizosphere another possible approach would be to utilize mutants with alterations specific for phenolic acids and/or compare a broader array of plant varieties with contrasting phenolic exudation profiles (Wu et al., 2001; Zwetsloot et al., 2018; Bergelson et al., 2019). Root exudates could also be compared and/or manipulated after extraction from roots and tested in simulated rhizosphere microcosms (de Vries et al., 2019).

Although phenolic acids had little effect on the total fungal biomass, fungal community analysis for three phenolic acids revealed that they promoted specific genera, when added in combination with primary root metabolites. The analyzed phenolic acids increased the relative abundance of *Fusarium*, *Trichoderma* and *Fusicolla* spp., as compared to primary metabolites alone. Both *Fusarium* and *Trichoderma* comprise soil-borne fungi able to invade root internal tissues (Chen et al., 2018). Little information is available about *Fusicolla*, however, the close relationship of this genus with *Fusarium* suggests they could share the ability to reach the plant interior (Gräfenhan et al., 2011). Catabolism of phenolic acids, in particular of salicylic acid, is required by endophytic fungi in order to cope with plant immune responses in internal plant tissues (Qi et al., 2012) which could give a competitive advantage to these fungal groups also in the rhizosphere. The ability of *Fusarium* spp. to grow in presence of low levels of phenolic acids has been documented (Targoński et al., 1986; Chen et al., 2018). Similarly, *Trichoderma* spp. abundance in the rhizosphere was found to be promoted by vanillic acid in earlier studies (Chen et al., 2018; Zhou and Wu, 2018). Earlier research has indicated the attraction of rhizosphere- and root-inhabiting bacteria by phenolic acids (Li et al., 2012; Badri et al., 2013). Moreover, stress-induced changes in root exudation were also associated with an selective chemoattraction of *Trichoderma* (Lombardi et al., 2018). Our results indicate that phenolic root exudates could play a role in stress-induced attraction of endophytic fungi.

In this study it was not possible to determine if *Fusarium* spp. belonged to pathogenic or non-pathogenic guilds, as they were solely identified based on ITS sequences, that is not a good marker for *Fusaria*. *Trichoderma* spp. are well-known as beneficial facultative endophytes of plants (Kepler et al., 2017; Chen et al., 2018). With regard to *Fusicolla* spp., there is little information on their virulence to plants (Gräfenhan et al., 2011). This potential variety of fungal guilds promoted by phenolic acids is consistent with the evidence that phenolic acid degradation is a virulence factor found in pathogenic fungi, but that mutualistic soil-borne fungi also share this trait (Lahrmann et al., 2015). Hence, phenolic acids appear to affect both non-pathogenic and pathogenic fungal endophytes. Further analyses targeting activity and gene expression, rather than (relative) abundances, are required to get more insight in the response of potential fungal endophytes to phenolic root exudates.

Besides modulating the fungal community composition, the analyzed phenolic acids also affected bacteria in the simulated rhizosphere. Bacteria belonging to all the most abundant classes in soils (i.e., α-, γ-Proteobacteria, Bacilli and Clostridia) were overrepresented in the rhizosphere receiving salicylic acid and primary metabolites, as compared to primary metabolites alone. At the same time, other members of the same classes were underrepresented in the soil receiving salicylic acid and primary metabolites. As indicated above, phenolic acids are known to exert a strong selection on rhizosphere bacterial communities (Pumphrey and Madsen, 2008; Li et al., 2012; Badri et al., 2013; Zhalnina et al., 2018). They favor those groups that possess the specialized metabolism for phenolic degradation, while acting as a deterrent for those sensitive to phenolic acids at relatively low concentrations (Blum and Shafer, 1988; Fierer et al., 2005; Pumphrey and Madsen, 2008). Similar to this study, Lebeis et al. (2015) observed opposing effects among taxonomically closely related bacteria.

### Stimulation Fungal Biomass and Decreased Bacterial Numbers by Salicylic Acid

Salicylic acid alone did not increase fungal or bacterial abundance in the simulated rhizosphere. However, salicylic acid combined with primary metabolites increased fungal biomass and decreased bacterial numbers, hence shifting the balance between the two groups. On the other hand, *A. thaliana sid2* plants impaired in the production of salicylic acid harbored similar fungal biomass in their rhizospheres as the wild type. The fungal-stimulating effect observed in the model rhizosphere could be a consequence of a better ability of some fungi to utilize this plant hormone for growth, resulting in a competitive advantage of fungi, as compared to bacteria. Lebeis et al. (2015) showed that salicylic acid and its downstream cascade is not only an important regulator of the plant immune system, but is also a modulator of the root bacterial community composition, as it selectively promoted specific bacterial families, whilst inhibiting others. To our knowledge, saprotrophic fungi have rarely been included in studies on the effect of salicylic acid on root and rhizosphere microbiomes. Our result points at an as-yet-undefined role of salicylic acid in modulating fungal abundances in the rhizosphere. In particular, *Trichoderma* and *Fusarium* spp. are triggered by the combination of salicylic acid in a background of primary metabolites.

Yet, fungal biomass was not affected by the knockdown of salicylic acid synthesis in *sid2 A. thaliana* plants. Therefore, it is not clear whether the fungus-stimulating effect of salicylic acid observed in the rhizosphere simulation microcosm is also occurring *in planta*. Since root exudate composition was not determined, it is not possible to validate if the lack of response of fungi was indeed occurring despite different concentrations of salicylic acid. Lack of fungal biomass stimulation can be affected by the fact that a large difference in salicylic acid accumulation is seen between *sid2* and wild-type plants only after pathogen infection, whereas such a difference is less marked during constitutive salicylic acid biosynthesis (Wildermuth et al., 2001). Moreover, *sid2* plants could partially compensate for the isochorismate synthase knockdown mutation by the upregulation of alternate pathways for salicylic acid production. Considering the limitations of this approach, and apparent contrasting results in the two experiments, it will be of interest to further address whether salicylic acid operates as a modulator of size, composition and activity of the rhizosphere fungal community.

## Conclusion and Perspectives

Our research indicates that phenolic acids, at the concentrations found in root exudates, have little effect on the biomass of saprotrophic fungi inhabiting the rhizosphere. Therefore, selecting crop varieties with a higher exudation of phenolic acids probably does not represent an effective strategy for increasing fungal abundance in the rhizosphere of crop plants growing in fungal-poor arable soils. Nevertheless, this study shows that phenolic acids act as modulators of fungal communities by attracting soil-borne fungal endophytes. These potentially belonged to both pathogenic and mutualistic groups. Further research should indicate if this endophyte-attraction aspect of phenolic acids in root exudates has potential for steering toward improved functioning of rhizosphere- and root microbial communities.

## Data Availability Statement

The datasets presented in this study are publicly available at Dryad doi: 10.5061/dryad.573n5tb6z and at the European Nucleotide Archive (sequencing data): accession PRJEB38475(ERP121911).

## Author Contributions

AC, SEH, and WB conceived and planned the experiments. AC, MB, and MPJH executed the experiments. AC analyzed the data and wrote the manuscript in consultation with SEH and WB. All authors contributed to the article and approved the submitted version.

## Conflict of Interest

The authors declare that the research was conducted in the absence of any commercial or financial relationships that could be construed as a potential conflict of interest.

## Supplementary Material

Supplementary Figure 1 Effect of phenolic acids on fungal biomass, as measured in the upper sterile sand compartment of rhizosphere simulation microcosms. Phenolic acids were vanillic acid (Van), syringic acid (Syr), gallic acid (Gal), salicylic acid (Sal), chlorogenic acid (Chl), nicotinic acid (Nic), ferulic acid (Fer) and cinnamic acid (Cin). Phenolic acids were added alone or with a background of soluble primary metabolites (PM) (^∙^0.1 > *p* > 0.05, *0.05 > *p* > 0.01, **0.01 > *p* > 0.001, ****p* < 0.001).

Supplementary Figure 2 Effect of phenolic acids (Van = vanillic acid, Sal = salicylic acid, Chl = chlorogenic acid) alone and combined with primary metabolites (PM) on α-diversity of the soil fungal **(A,B)** and bacterial community **(C,D)** as measured in rhizosphere simulation microcosms. Alpha diversity is shown by Chao1 **(A,C)** and Shannon indexes **(B,D)**. The main effect of PM and the effect of individual phenolic acids are shown (** 0,01 > *p* > 0,001, *** *p* < 0,001).

Supplementary Figure 3 Effect of salicylic acid + PM as compared to PM only on bacterial taxa in the arable soil layer of rhizosphere simulation microcosms. Bacterial SVs over- (log_2_ fold change > 0) and under-represented (log_2_ fold change < 0) in soil treated with salicylic acid + PM were highlighted by differential abundance analysis (wald test, *p* < 0,01). Bacterial SVs are grouped by class and bacterial families are specified for the seven most abundant bacterial classes (δ-, γ- and α-Proteobacteria, Bacilli, Clostridia, Bacteroidia and Actinobacteria). SVs belonging to classes with < 2% relative abundance are classified as “Other.”

Supplementary Table 1 Summary of ANOVA results on ergosterol **(A)** and 16S copy number **(B)** as a function of the type of phenolic acid, compartment (soil/sand), and presence or absence of primary metabolites (PM) (Exp. 1). *df*.: degrees of freedon, *SS*: sum of squares, *MS*: mean sum of squares.

Supplementary Table 2 Summary of ANOVA results on ergosterol as a function of the *Arabidopsis* line (*A.t.* line) and developmental stage (dev. stage) (Exp. 2), shown for ergosterol in the rhizosphere **(A)** and for the ergosterol increase in the rhizosphere from its concentration in the bulk soil **(B)**. *df*: degrees of freedom, *SS*: sum of squares, *MS*: mean sum of squares.

Supplementary Table 3 Summary of permutational analysis of multivariate variance (PERMANOVA) applied to fungal **(A)** and bacterial **(B)** community data of the arable soil layer in rhizosphere simulation microcosms using Bray-Curtis distances. The PERMANOVA was applied for the total dataset for partitioning the variance according to two factors (type of phenolic acid x presence of additional primary metabolites, PM). The effect of type of phenolic acid was also tested alone on two subsets of data (Phenolic acid with/without PM), as these had a better homogeneity of multivariate variance. *df*: degrees of freedom, *SS*: sum of squares *MS*: mean sum of squares, *p* values based on 999 permutations.

## References

[B1] Abarenkov K. Henrik Nilsson R. Larsson K.-H. Alexander I. J. Eberhardt U. Erland S. (2010). The UNITE database for molecular identification of fungi – recent updates and future perspectives. *New Phytol.* 186 281–285. 10.1111/j.1469-8137.2009.03160.x 20409185

[B2] Anderson M. J. Walsh D. C. I. (2013). PERMANOVA, ANOSIM, and the Mantel test in the face of heterogeneous dispersions: what null hypothesis are you testing? *Ecol. Monogr.* 83 557–574. 10.1890/12-2010.1

[B3] Arcand M. M. Helgason B. L. Lemke R. L. (2016). Microbial crop residue decomposition dynamics in organic and conventionally managed soils. *Appl. Soil Ecol.* 107 347–359. 10.1016/j.apsoil.2016.07.001

[B4] Badri D. V. Chaparro J. M. Zhang R. Shen Q. Vivanco J. M. (2013). Application of natural blends of phytochemicals derived from the root exudates of *Arabidopsis* to the soil reveal that phenolic-related compounds predominantly modulate the soil microbiome. *J. Biol. Chem.* 288 30503–30503. 10.1074/jbc.A112.433300 PMC3576057 23293028

[B5] Badri D. V. Quintana N. El Kassis E. G. Kim H. K. Choi Y. H. Sugiyama A. (2009). An ABC transporter mutation alters root exudation of phytochemicals that orovoke an overhaul of natural soil microbiota. *Plant Physiol.* 151 2006. 10.1104/pp.109.147462 19854857 PMC2785968

[B6] Bergelson J. Mittelstrass J. Horton M. W. (2019). Characterizing both bacteria and fungi improves understanding of the *Arabidopsis* root microbiome. *Sci. Rep.* 9:24. 10.1038/s41598-018-37208-z 30631088 PMC6328596

[B7] Blum U. Shafer S. R. (1988). Microbial populations and phenolic acids in soil. *Soil Biol. Biochem.* 20 793–800. 10.1016/0038-0717(88)90084-3

[B8] Brant J. B. Sulzman E. W. Myrold D. D. (2006). Microbial community utilization of added carbon substrates in response to long-term carbon input manipulation. *Soil Biol. Biochem.* 38 2219–2232. 10.1016/j.soilbio.2006.01.022

[B9] Buée M. De Boer W. Martin F. van Overbeek L. Jurkevitch E. (2009). The rhizosphere zoo: an overview of plant-associated communities of microorganisms, including phages, bacteria, archaea, and fungi, and of some of their structuring factors. *Plant Soil* 321 189–212. 10.1007/s11104-009-9991-3

[B10] Cain R. B. Bilton R. F. Darrah J. A. (1968). The metabolism of aromatic acids by micro-organisms. Metabolic pathways in the fungi. *Biochem. J.* 108 797–828. 10.1042/bj1080797 5691754 PMC1198887

[B11] Callahan B. J. McMurdie P. J. Rosen M. J. Han A. W. Johnson A. J. A. Holmes S. P. (2016). DADA2: high-resolution sample inference from Illumina amplicon data. *Nat. Methods* 13 581–583. 10.1038/nmeth.3869 27214047 PMC4927377

[B12] Caporaso J. G. Lauber C. L. Walters W. A. Berg-Lyons D. Lozupone C. A. Turnbaugh P. J. (2011). Global patterns of 16S rRNA diversity at a depth of millions of sequences per sample. *Proc. Natl. Acad. Sci. U.S.A.* 108(Suppl. 1) 4516–4522. 10.1073/pnas.1000080107 20534432 PMC3063599

[B13] Chaparro J. M. Badri D. V. Bakker M. G. Sugiyama A. Manter D. K. Vivanco J. M. (2013). Root exudation of phytochemicals in Arabidopsis follows specific patterns that are developmentally programmed and correlate with soil microbial functions. *PLoS One* 8:e55731. 10.1371/journal.pone.0055731 23383346 PMC3562227

[B14] Chen S. Yu H. Zhou X. Wu F. (2018). Cucumber (*Cucumis sativus* L.) seedling rhizosphere *Trichoderma* and *Fusarium* spp. communities altered by vanillic acid. *Front. Microbiol.* 9:2195. 10.3389/fmicb.2018.02195 30283420 PMC6157394

[B15] Clocchiatti A. Hannula S. E. van den Berg M. Korthals G. de Boer W. (2020). The hidden potential of saprotrophic fungi in arable soil: Patterns of short-term stimulation by organic amendments. *Appl. Soil Ecol.* 147:103434. 10.1016/j.apsoil.2019.103434

[B16] de Boer W. Hundscheid M. P. J. Klein Gunnewiek P. J. A. de Ridder-Duine A. S. Thion C. van Veen J. A. (2015). Antifungal rhizosphere bacteria can increase as response to the presence of saprotrophic fungi. *PLoS One* 10:e0137988. 10.1371/journal.pone.0137988 26393509 PMC4578881

[B17] de Ridder-Duine A. S. Smant W. van der Wal A. van Veen J. A. de Boer W. (2006). Evaluation of a simple, non-alkaline extraction protocol to quantify soil ergosterol. *Pedobiologia* 50 293–300. 10.1016/j.pedobi.2006.03.004

[B18] de Vries F. T. Bardgett R. D. (2012). Plant–microbial linkages and ecosystem nitrogen retention: lessons for sustainable agriculture. *Front. Ecol. Environ.* 10:425–432. 10.1890/110162

[B19] de Vries F. T. Williams A. Stringer F. Willcocks R. McEwing R. Langridge H. (2019). Changes in root-exudate-induced respiration reveal a novel mechanism through which drought affects ecosystem carbon cycling. *New Phytol.* 224 132–145. 10.1111/nph.16001 31218693 PMC6771481

[B20] Dennis P. G. Miller A. J. Hirsch P. R. (2010). Are root exudates more important than other sources of rhizodeposits in structuring rhizosphere bacterial communities? *FEMS Microbiol. Ecol.* 72 313–327. 10.1111/j.1574-6941.2010.00860.x 20370828

[B21] Deveau A. Bonito G. Uehling J. Paoletti M. Becker M. Bindschedler S. (2018). Bacterial–fungal interactions: ecology, mechanisms and challenges. *FEMS Microbiol. Rev.* 42 335–352. 10.1093/femsre/fuy008 29471481

[B22] di Lonardo D. P. de Boer W. Klein Gunnewiek P. J. A. Hannula S. E. van der Wal A. (2017). Priming of soil organic matter: chemical structure of added compounds is more important than the energy content. *Soil Biol. Biochem.* 108 41–54. 10.1016/j.soilbio.2017.01.017

[B23] Djajakirana G. Joergensen R. G. Meyer B. (1996). Ergosterol and microbial biomass relationship in soil. *Biol. Fertil. Soils* 22 299–304. 10.1007/BF00334573

[B24] Duah-Yentumi S. Johnson D. B. (1986). Changes in soil microflora in response to repeated applications of some pesticides. *Soil Biol. Biochem.* 18 629–635. 10.1016/0038-0717(86)90086-6

[B25] Fierer N. Jackson J. A. Vilgalys R. Jackson R. B. (2005). Assessment of soil microbial community structure by use of taxon-specific quantitative PCR assays. *Appl. Environ. Microbiol.* 71:4117. 10.1128/AEM.71.7.4117-4120.2005 16000830 PMC1169028

[B26] Gajendiran A. Krishnamoorthy S. Abraham J. (2016). Microbial degradation of low-density polyethylene (LDPE) by *Aspergillus clavatus* strain JASK1 isolated from landfill soil. *3 Biotech* 6:52. 10.1007/s13205-016-0394-x 28330123 PMC4752946

[B27] Gao X. Zhang S. Zhao X. Wu Q. (2018). Potassium-induced plant resistance against soybean cyst nematode via root exudation of phenolic acids and plant pathogen-related genes. *PLoS One* 13:e0200903. 10.1371/journal.pone.0200903 30059518 PMC6066213

[B28] Gräfenhan T. Schroers H.-J. Nirenberg H. I. Seifert K. A. (2011). An overview of the taxonomy, phylogeny, and typification of nectriaceous fungi in *Cosmospora*, *Acremonium*, *Fusarium*, *Stilbella*, and *Volutella*. *Stud. Mycol.* 68 79–113. 10.3114/sim.2011.68.04 21523190 PMC3065986

[B29] Gransee A. Wittenmayer L. (2000). Qualitative and quantitative analysis of water-soluble root exudates in relation to plant species and development. *J. Plant Nutr. Soil Sci.* 163 381–385.

[B30] Griffiths B. S. Ritz K. Ebblewhite N. Dobson G. (1998). Soil microbial community structure: effects of substrate loading rates. *Soil Biol. Biochem.* 31 145–153. 10.1016/S0038-0717(98)00117-5

[B31] Hannula S. E. Boschker H. T. S. de Boer W. van Veen J. A. (2012). 13C pulse-labeling assessment of the community structure of active fungi in the rhizosphere of a genetically starch-modified potato (*Solanum tuberosum*) cultivar and its parental isoline. *New Phytol.* 194 784–799. 10.1111/j.1469-8137.2012.04089.x 22413848

[B32] Hannula S. E. de Boer W. van Veen J. A. (2010). In situ dynamics of soil fungal communities under different genotypes of potato, including a genetically modified cultivar. *Soil Biol. Biochem.* 42 2211–2223. 10.1016/j.soilbio.2010.08.020

[B33] Hugoni M. Luis P. Guyonnet J. Haichar F. el Z (2018). Plant host habitat and root exudates shape fungal diversity. *Mycorrhiza* 28 451–463. 10.1007/s00572-018-0857-5 30109473

[B34] Hünninghaus M. Dibbern D. Kramer S. Koller R. Pausch J. Schloter-Hai B. (2019). Disentangling carbon flow across microbial kingdoms in the rhizosphere of maize. *Soil Biol. Biochem.* 134 122–130. 10.1016/j.soilbio.2019.03.007

[B35] Iannucci A. Fragasso M. Platani C. Papa R. (2013). Plant growth and phenolic compounds in the rhizosphere soil of wild oat (*Avena fatua* L.). *Front. Plant Sci.* 4:509. 10.3389/fpls.2013.00509 24381576 PMC3865607

[B36] Ihrmark K. Bödeker I. T. M. Cruz-Martinez K. Friberg H. Kubartova A. Schenck J. (2012). New primers to amplify the fungal ITS2 region – evaluation by 454-sequencing of artificial and natural communities. *FEMS Microbiol. Ecol.* 82 666–677. 10.1111/j.1574-6941.2012.01437.x 22738186

[B37] Kazerooni E. A. Maharachchikumbura S. S. N. Rethinasamy V. Al-Mahrouqi H. Al-Sadi A. M. (2017). Fungal diversity in tomato rhizosphere soil under conventional and desert farming systems. *Front. Microbiol.* 8:1462. 10.3389/fmicb.2017.01462 28824590 PMC5539375

[B38] Kepler R. M. Maul J. E. Rehner S. A. (2017). Managing the plant microbiome for biocontrol fungi: examples from Hypocreales. *Curr. Opin. Microbiol.* 37 48–53. 10.1016/j.mib.2017.03.006 28441534

[B39] Kohler J. Caravaca F. Carrasco L. Roldán A. (2007). Interactions between a plant growth-promoting rhizobacterium, an AM fungus and a phosphate-solubilising fungus in the rhizosphere of Lactuca sativa. *Appl. Soil Ecol.* 35 480–487. 10.1016/j.apsoil.2006.10.006

[B40] Koike N. Hyakumachi M. Kageyama K. Tsuyumu S. Doke N. (2001). Induction of systemic resistance in cucumber against several diseases by plant growth-promoting fungi: lignification and superoxide generation. *Eur. J. Plant Pathol.* 107 523–533. 10.1023/A:1011203826805

[B41] Lahrmann U. Strehmel N. Langen G. Frerigmann H. Leson L. Ding Y. (2015). Mutualistic root endophytism is not associated with the reduction of saprotrophic traits and requires a noncompromised plant innate immunity. *New Phytol.* 207 841–857. 10.1111/nph.13411 25919406

[B42] Latz M. A. C. Jensen B. Collinge D. B. Jørgensen H. J. L. (2018). Endophytic fungi as biocontrol agents: elucidating mechanisms in disease suppression. *Plant Ecol. Divers.* 11 555–567. 10.1080/17550874.2018.1534146

[B43] Lebeis S. L. Paredes S. H. Lundberg D. S. Breakfield N. Gehring J. McDonald M. (2015). Salicylic acid modulates colonization of the root microbiome by specific bacterial taxa. *Science* 349:860. 10.1126/science.aaa8764 26184915

[B44] Li P. Ma L. Feng Y. L. Mo M. H. Yang F. X. Dai H. F. (2012). Diversity and chemotaxis of soil bacteria with antifungal activity against *Fusarium* wilt of banana. *J. Ind. Microbiol. Biotechnol.* 39 1495–1505. 10.1007/s10295-012-1163-4 22763749

[B45] Lombardi N. Vitale S. Turrà D. Reverberi M. Fanelli C. Vinale F. (2018). Root exudates of stressed plants stimulate and attract trichoderma soil fungi. *Mol. Plant Microbe Interact.* 31 982–994. 10.1094/MPMI-12-17-0310-R 29547355

[B46] Lucas S. T. D’Angelo E. M. Williams M. A. (2014). Improving soil structure by promoting fungal abundance with organic soil amendments. *Appl. Soil Ecol.* 75 13–23. 10.1016/j.apsoil.2013.10.002

[B47] Mäkelä M. R. Marinoviæ M. Nousiainen P. Liwanag A. J. M. Benoit I. Sipilä J. (2015). “Aromatic metabolism of filamentous fungi in relation to the presence of aromatic compounds in plant biomass,” in *Advances in Applied Microbiology*, eds Sariaslani S. Gadd G. M. (Cambridge, MA: Academic Press), 63–137. 10.1016/bs.aambs.2014.12.001 25911233

[B48] Narasimhan K. Basheer C. Bajic V. B. Swarup S. (2003). Enhancement of plant-microbe interactions using a rhizosphere metabolomics-driven approach and its application in the removal of polychlorinated biphenyls. *Plant Physiol.* 132 146–153. 10.1104/pp.102.016295 12746520 PMC166960

[B49] Naznin H. A. Kiyohara D. Kimura M. Miyazawa M. Shimizu M. Hyakumachi M. (2014). Systemic resistance induced by volatile organic compounds emitted by plant growth-promoting fungi in *Arabidopsis thaliana*. *PLoS One* 9:e86882. 10.1371/journal.pone.0086882 24475190 PMC3903595

[B50] Nguyen N. H. Song Z. Bates S. T. Branco S. Tedersoo L. Menke J. (2016). FUNGuild: an open annotation tool for parsing fungal community datasets by ecological guild. *Fungal Ecol.* 20 241–248. 10.1016/j.funeco.2015.06.006

[B51] Pausch J. Kramer S. Scharroba A. Scheunemann N. Butenschoen O. Kandeler E. (2016). Small but active – pool size does not matter for carbon incorporation in below-ground food webs. *Funct. Ecol.* 30 479–489. 10.1111/1365-2435.12512

[B52] Pausch J. Kuzyakov Y. (2018). Carbon input by roots into the soil: quantification of rhizodeposition from root to ecosystem scale. *Glob. Change Biol.* 24 1–12. 10.1111/gcb.13850 28752603

[B53] Porras-Alfaro A. Herrera J. Natvig D. O. Lipinski K. Sinsabaugh R. L. (2011). Diversity and distribution of soil fungal communities in a semiarid grassland. *Mycologia* 103 10–21. 10.3852/09-297 20943560

[B54] Pumphrey G. M. Madsen E. L. (2008). Field-based stable isotope probing reveals the identities of benzoic acid-metabolizing microorganisms and their in situ growth in agricultural soil. *Appl. Environ. Microbiol.* 74 4111–4118. 10.1128/AEM.00464-08 18469130 PMC2446519

[B55] Punja Z. K. Utkhede R. S. (2003). Using fungi and yeasts to manage vegetable crop diseases. *Trends Biotechnol.* 21 400–407. 10.1016/S0167-7799(03)00193-8 12948673

[B56] Qi P.-F. Johnston A. Balcerzak M. Rocheleau H. Harris L. J. Long X.-Y. (2012). Effect of salicylic acid on *Fusarium graminearum*, the major causal agent of fusarium head blight in wheat. *Fungal Biol.* 116 413–426. 10.1016/j.funbio.2012.01.001 22385623

[B57] Qin S. Yeboah S. Xu X. Liu Y. Yu B. (2017). Analysis on fungal diversity in rhizosphere soil of continuous cropping potato subjected to different furrow-ridge mulching managements. *Front. Microbiol.* 8:845. 10.3389/fmicb.2017.00845 28539923 PMC5423957

[B58] Quist C. W. Schrama M. de Haan J. J. Smant G. Bakker J. van der Putten W. H. (2016). Organic farming practices result in compositional shifts in nematode communities that exceed crop-related changes. *Appl. Soil Ecol.* 98 254–260. 10.1016/j.apsoil.2015.10.022

[B59] Ray S. Mishra S. Bisen K. Singh S. Sarma B. K. Singh H. B. (2018). Modulation in phenolic root exudate profile of *Abelmoschus esculentus* expressing activation of defense pathway. *Microbiol. Res.* 207 100–107. 10.1016/j.micres.2017.11.011 29458844

[B60] Rivers A. Weber K. Gardner T. Liu S. Armstrong S. (2018). ITSxpress: software to rapidly trim internally transcribed spacer sequences with quality scores for marker gene analysis. *F1000Research* 7:1418. 10.12688/f1000research.15704.1 30416717 PMC6206612

[B61] Saldajeno M. G. B. Chandanie W. A. Kubota M. Hyakumachi M. (2008). “Effects of interactions of arbuscular mycorrhizal fungi and beneficial saprophytic mycoflora on plant growth and disease protection,” in *Mycorrhizae: Sustainable Agriculture and Forestry*, eds Siddiqui Z. A. Akhtar M. S. Futai K. (Dordrecht: Springer Netherlands), 211–226. 10.1007/978-1-4020-8770-7_9

[B62] Sampedro I. Aranda E. Martıìn J. Garcıìa-Garrido J. M. Garcıìa-Romera I. Ocampo J. A. (2004). Saprobic fungi decrease plant toxicity caused by olive mill residues. *Appl. Soil Ecol.* 26 149–156. 10.1016/j.apsoil.2003.10.011

[B63] Shalaby S. Horwitz B. A. (2015). Plant phenolic compounds and oxidative stress: integrated signals in fungal–plant interactions. *Curr. Genet.* 61 347–357. 10.1007/s00294-014-0458-6 25407462

[B64] Shao H. Zhang Y. (2017). Non-target effects on soil microbial parameters of the synthetic pesticide carbendazim with the biopesticides cantharidin and norcantharidin. *Sci. Rep.* 7:5521. 10.1038/s41598-017-05923-8 28717209 PMC5514074

[B65] Shukla K. Sharma S. Singh N. Singh V. Tiwari K. Singh S. (2011). Nature and role of root exudates: efficacy in bioremediation. *Afr. J. Biotechnol.* 10 9717–9724. 10.5897/AJB10.2552

[B66] Šidák Z. (1967). Rectangular confidence regions for the means of multivariate normal distributions. *J. Am. Stat. Assoc.* 62 626–633. 10.1080/01621459.1967.10482935

[B67] Suseela V. Alpert P. Nakatsu C. H. Armstrong A. Tharayil N. (2016). Plant–soil interactions regulate the identity of soil carbon in invaded ecosystems: implication for legacy effects. *Funct. Ecol.* 30 1227–1238. 10.1111/1365-2435.12591

[B68] Targoński Z. Rogalski J. Szczodrak J. (1986). Decomposition of 14C-labelled vanillic acid and its related compounds by *Fusarium oxysporum*. *Syst. Appl. Microbiol.* 8 148–151. 10.1016/S0723-2020(86)80163-1

[B69] Tavi N. M. Martikainen P. J. Lokko K. Kontro M. Wild B. Richter A. (2013). Linking microbial community structure and allocation of plant-derived carbon in an organic agricultural soil using 13CO2 pulse-chase labelling combined with 13C-PLFA profiling. *Soil Biol. Biochem.* 58 207–215. 10.1016/j.soilbio.2012.11.013

[B70] van der Putten W. H. Bradford M. A. Pernilla Brinkman E. van de Voorde T. F. J. Veen G. F. (2016). Where, when and how plant–soil feedback matters in a changing world. *Funct. Ecol.* 30 1109–1121. 10.1111/1365-2435.12657

[B71] van der Wal A. van Veen J. A. Pijl A. Summerbell R. de Boer W. (2006). Constraints on development of fungal biomass and decomposition processes during restoration of arable sandy soils. *Soil Biol. Biochem.* 38 2890–2902. 10.1016/j.soilbio.2006.04.046

[B72] Waldrop M. P. Firestone M. K. (2004). Microbial community utilization of recalcitrant and simple carbon compounds: impact of oak-woodland plant communities. *Oecologia* 138 275–284. 10.1007/s00442-003-1419-9 14614618

[B73] Wang Y. Li C. Wang Q. Wang H. Duan B. Zhang G. (2016). Environmental behaviors of phenolic acids dominated their rhizodeposition in boreal poplar plantation forest soils. *J. Soils Sediments* 16 1858–1870. 10.1007/s11368-016-1375-8

[B74] Wildermuth M. C. Dewdney J. Wu G. Ausubel F. M. (2001). Isochorismate synthase is required to synthesize salicylic acid for plant defence. *Nature* 414 562–565. 10.1038/35107108 11734859

[B75] Wu H. Haig T. Pratley J. Lemerle D. An M. (2001). Allelochemicals in wheat (*Triticum aestivum* L.): cultivar difference in the exudation of phenolic acids. *J. Agric. Food Chem.* 49 3742–3745. 10.1021/jf010111x 11513658

[B76] Xia Y. Sahib M. R. Amna A. Opiyo S. O. Zhao Z. Gao Y. G. (2019). Culturable endophytic fungal communities associated with plants in organic and conventional farming systems and their effects on plant growth. *Sci. Rep.* 9:1669. 10.1038/s41598-018-38230-x 30737459 PMC6368545

[B77] Xiong W. Li R. Ren Y. Liu C. Zhao Q. Wu H. (2017). Distinct roles for soil fungal and bacterial communities associated with the suppression of vanilla *Fusarium* wilt disease. *Soil Biol. Biochem.* 107 198–207. 10.1016/j.soilbio.2017.01.010

[B78] Yadav J. Verma J. Tiwari K. (2011). Plant growth promoting activities of fungi and their effect on chickpea plant growth. *Asian J. Biol. Sci.* 4 291–299. 10.3923/ajbs.2011.291.299

[B79] Zhalnina K. Louie K. B. Hao Z. Mansoori N. da Rocha U. N. Shi S. (2018). Dynamic root exudate chemistry and microbial substrate preferences drive patterns in rhizosphere microbial community assembly. *Nat. Microbiol.* 3 470–480. 10.1038/s41564-018-0129-3 29556109

[B80] Zhou X. Wu F. (2013). Artificially applied vanillic acid changed soil microbial communities in the rhizosphere of cucumber (*Cucumis sativus* L.). *Can. J. Soil Sci.* 93 13–21. 10.4141/cjss2012-039

[B81] Zhou X. Wu F. (2018). Vanillic acid changed cucumber (*Cucumis sativus* L.) seedling rhizosphere total bacterial, *Pseudomonas* and *Bacillus* spp. communities. *Sci. Rep.* 8:4929. 10.1038/s41598-018-23406-2 29563548 PMC5862977

[B82] Zhou X. Yu G. Wu F. (2012). Soil phenolics in a continuously mono-cropped cucumber (*Cucumis sativus* L.) system and their effects on cucumber seedling growth and soil microbial communities. *Eur. J. Soil Sci.* 63 332–340. 10.1111/j.1365-2389.2012.01442.x

[B83] Zwetsloot M. J. Kessler A. Bauerle T. L. (2018). Phenolic root exudate and tissue compounds vary widely among temperate forest tree species and have contrasting effects on soil microbial respiration. *New Phytol.* 218 530–541. 10.1111/nph.15041 29473651

[B84] Zwetsloot M. J. Ucros J. M. Wickings K. Wilhelm R. C. Sparks J. Buckley D. H. (2020). Prevalent root-derived phenolics drive shifts in microbial community composition and prime decomposition in forest soil. *Soil Biol. Biochem.* 145:107797. 10.1016/j.soilbio.2020.107797

