# Supplementary figures and images for: Evaluation of Phenolic Root Exudates as Stimulants of Saptrophic Fungi in the Rhizosphere
Source: Front Microbiol. 2021 Apr 14;12:644046. doi: 10.3389/fmicb.2021.644046 (PMC8079663; doi:10.3389/fmicb.2021.644046)

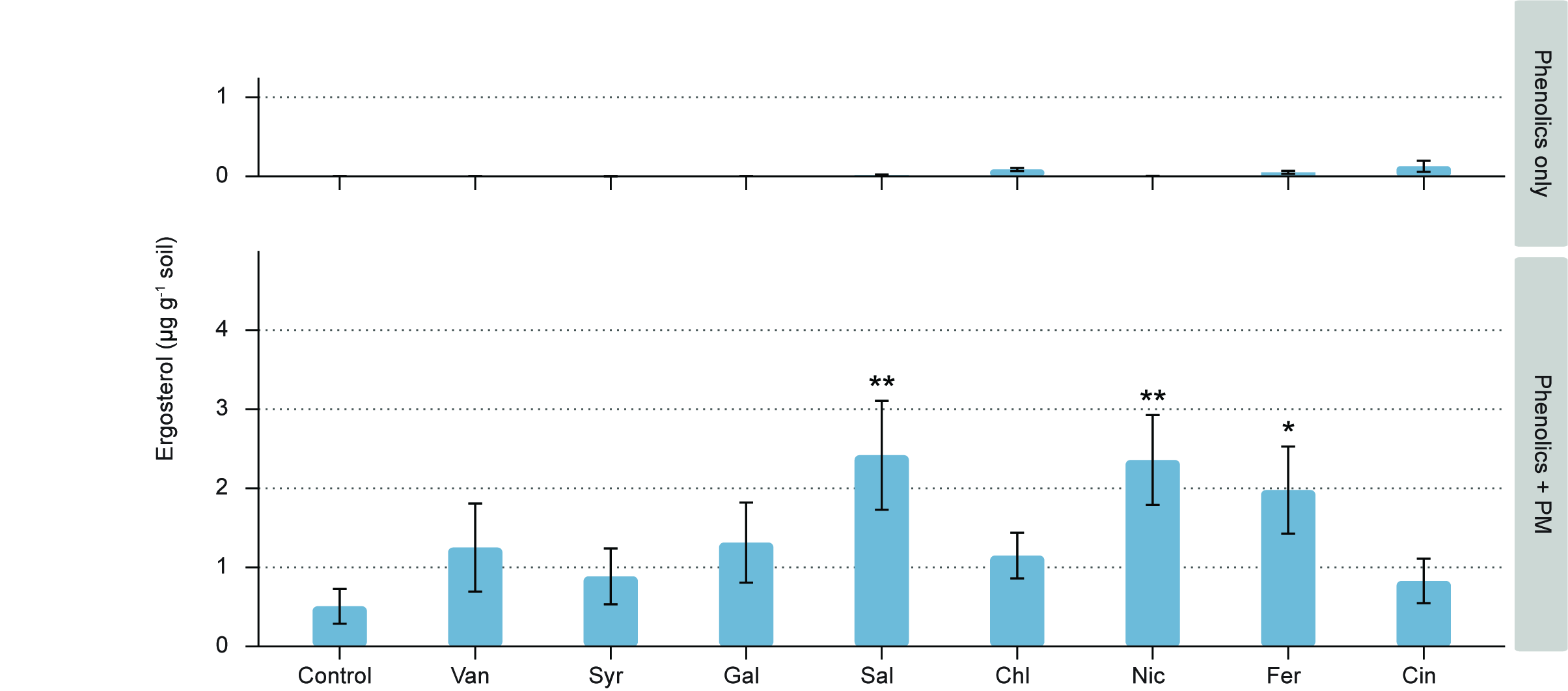

Supplement: Supplementary Figure 1 — Effect of phenolic acids on fungal biomass, as measured in the upper sterile sand compartment of rhizosphere simulation microcosms. Phenolic acids were vanillic acid (Van), syringic acid (Syr), gallic acid (Gal), salicylic acid (Sal), chlorogenic acid (Chl), nicotinic acid (Nic), ferulic acid (Fer) and cinnamic acid (Cin). Phenolic acids were added alone or with a background of soluble primary metabolites (PM) (∙0.1 > p > 0.05, *0.05 > p > 0.01, **0.01 > p > 0.001, ***p < 0.001). [file Image_1.TIF]

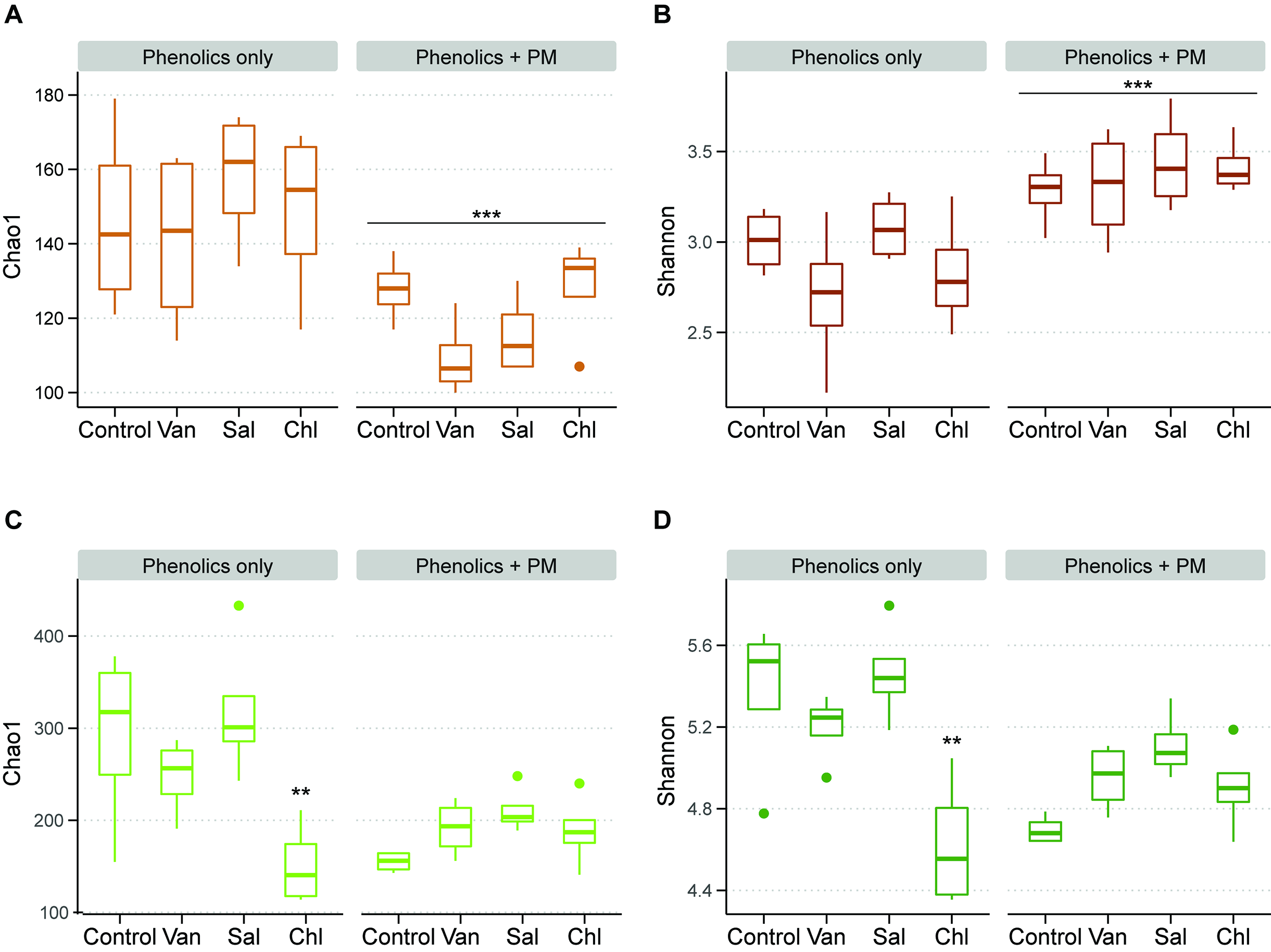

Supplement: Supplementary Figure 2 — Effect of phenolic acids (Van = vanillic acid, Sal = salicylic acid, Chl = chlorogenic acid) alone and combined with primary metabolites (PM) on α-diversity of the soil fungal (A,B) and bacterial community (C,D) as measured in rhizosphere simulation microcosms. Alpha diversity is shown by Chao1 (A,C) and Shannon indexes (B,D). The main effect of PM and the effect of individual phenolic acids are shown (** 0,01 > p > 0,001, *** p < 0,001). [file Image_2.TIF]

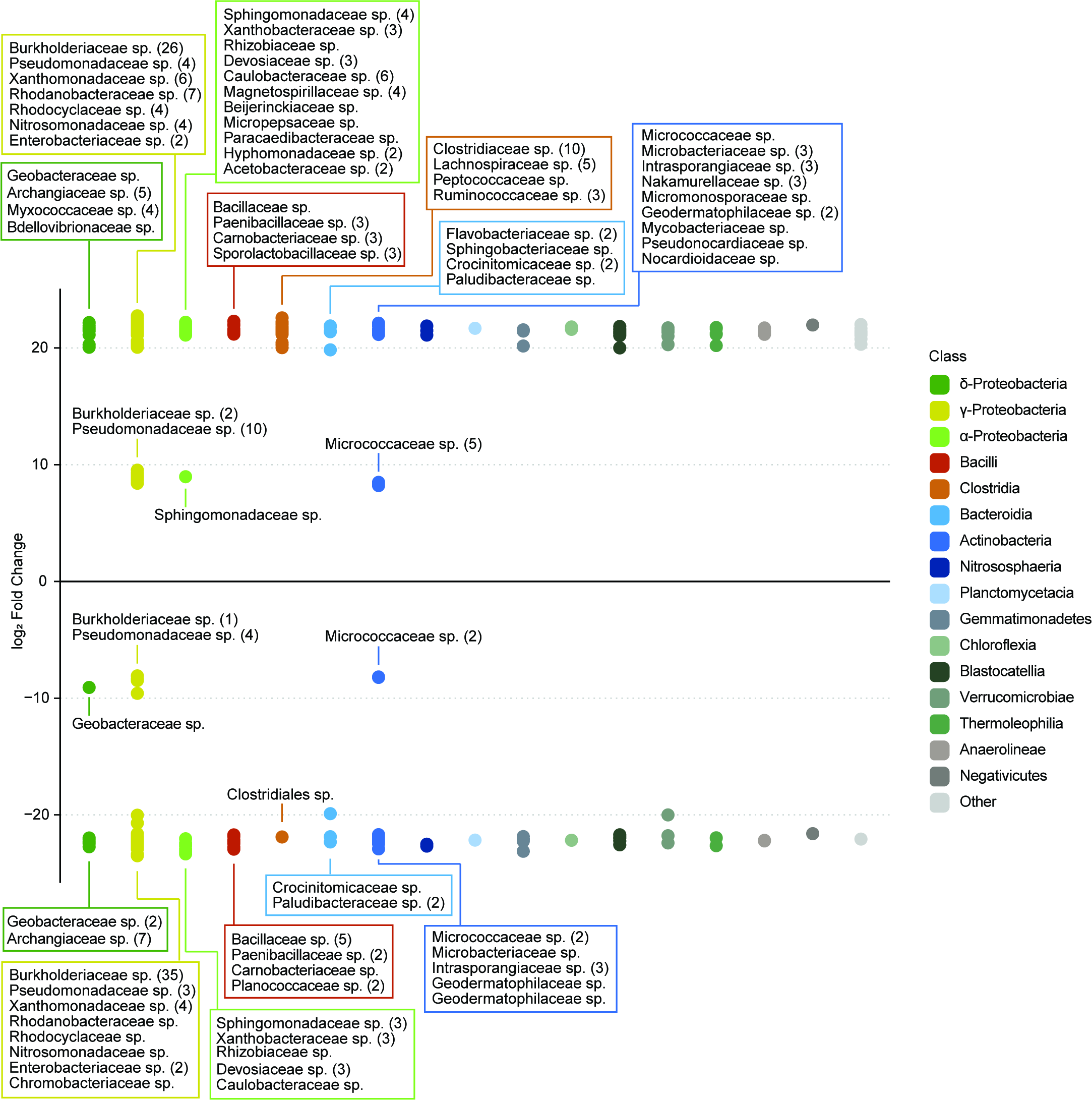

Supplement: Supplementary Figure 3 — Effect of salicylic acid + PM as compared to PM only on bacterial taxa in the arable soil layer of rhizosphere simulation microcosms. Bacterial SVs over- (log2 fold change > 0) and under-represented (log2 fold change < 0) in soil treated with salicylic acid + PM were highlighted by differential abundance analysis (wald test, p < 0,01). Bacterial SVs are grouped by class and bacterial families are specified for the seven most abundant bacterial classes (δ-, γ- and α-Proteobacteria, Bacilli, Clostridia, Bacteroidia and Actinobacteria). SVs belonging to classes with < 2% relative abundance are classified as “Other.” [file Image_3.TIF]
